# Monocyte and Macrophage Function Diversity

**DOI:** 10.3390/ijms232012404

**Published:** 2022-10-17

**Authors:** Malgorzata Kloc, Jacek Z. Kubiak

**Affiliations:** 1 Transplant Immunology, The Houston Methodist Research Institute, Houston, TX 77030, USA; 2 Department of Surgery, The Houston Methodist Hospital, Houston, TX 77030, USA; 3 Department of Genetics, The University of Texas, MD Anderson Cancer Center, Houston, TX 77030, USA; 4 Dynamics and Mechanics of Epithelia Group, Faculty of Medicine, Institute of Genetics and Development of Rennes, University of Rennes, CNRS, UMR 6290, 35000 Rennes, France; 5 Laboratory of Molecular Oncology and Innovative Therapies, Department of Oncology, Military Institute of Medicine, 04-141 Warsaw, Poland

In the last decade, there has been a tremendous revival of interest in monocyte and macrophages. In 2022 alone, over 500 papers about monocytes, and over 1600 about macrophages were listed in PubMed. Through the years, our concept and comprehension of monocytes and macrophages and their functions evolved from treating them as simple ameba-like cells whose only occupation was the phagocytosis of foreign objects and pathogens, to the extremely complicated, both phenotypically and functionally, cells. Macrophages affect nearly all processes of homeostasis, aging, and diseases, and are intertwined, by being both effector and signaling source, with the elaborate intercellular signaling pathways and a variety of cell types [1,2,3]. Another level of phenotypic and functional complexity comes into play when monocytes or macrophages fuse with each other or with different cells forming completely new entities with new or enhanced functions (Figure 1); [4,5].

Papers published in the IJMS Special Issue, “Human and Animal Monocytes and Macrophages in Homeostasis and Disease 3.0,” address some of the aspects of monocyte and macrophage complexity, expanding our knowledge on these amazing cells and suggesting novel macrophage-targeted therapies.

In their most primeval function, macrophages phagocytose and kill microbes, such as Gram-negative bacteria, which contain lipopolysaccharides (LPS) in their cell wall. Macrophages are well-suited for LPS detection because their surface is decorated with the pattern recognition receptors, such as toll-like receptor 4 (TLR-4), which recognizes molecules frequently found in microbes. In a healthy organism, various beneficial bacteria residing in the gut are kept from entering the blood by a barrier of tightly adhering cells of the intestinal wall. When this physical barrier is weakened, the so-called leaky gut becomes permeable allowing bacteria to enter the circulation, which leads to systemic inflammation. Thim-Uam et al. [6] describe how splenectomy- or LPS- induced gut barrier defect, which causes severe bacteremia followed by endotoxemia-induced LPS tolerance, affects macrophage functions. They found that in the state of LPS tolerance, macrophages have lower energy status, lower cytokine and pro-inflammatory molecules (iNOS and IL-1 β) production, but higher anti-inflammatory molecules (Arginase-1 and TGF- β). The authors concluded that in any post-splenectomy condition (surgery, accidents, diseases), the clinical intervention should target macrophages and try to manipulate their functions.

Macrophages are known to be major players in inflammatory diseases such as hypertension, which is associated with chronic inflammation. Bryniarski et al. [7] analyzed how anti-hypertension drugs, angiotensin-converting enzyme (ACE2) inhibitors, and angiotensin II receptor blockers reduce the inflammatory activity of macrophages and other immune cells. In very informative tables they listed all studied drugs and their effect on molecules related to the immune response and different immune cells. Macrophages are well-suited to respond to these drugs because they express a type 2 angiotensin II (Ang II) receptor (AT2R) and angiotensin converting enzyme 2 (ACE2) receptor. AT2R and ACE2 receptors initiate a signaling cascade in macrophages that modulate their activation and production of chemokines and cytokines. This is also very relevant to the recent COVID-19 pandemic because ACE2 receptors bind the SARS-CoV-2 Spike Protein, facilitating virus internalization and intercellular spreading [7,8]. The anti-hypertension drugs modulate immune response parameters not only in hypertensive but also in healthy patients; thus, their anti-inflammatory properties can be possibly used to treat other than hypertension inflammatory diseases including overreactive macrophages in viral infections.

Another group of inflammatory diseases involving various immune cells, including macrophages, are autoimmune diseases. Humans have over 80 types of autoimmune diseases, such as rheumatoid arthritis, ulcerative colitis, Crohn’s disease, dermatomyositis, systemic sclerosis, and lupus. Many autoimmune diseases exhibit inflammatory lung lesions and fibrosis, referred to as autoimmune lung disease. Eleva et al. [9] studied the effect of anti-inflammatory corticosteroid dexamethasone on lung injury in the autoimmune mouse model. Their study showed that dexamethasone ameliorates the severity of lung injury, partially through the polarization of macrophages toward the alternatively activated (anti-inflammatory) M2 macrophages. Such macrophages are involved in tissue regeneration and repair. These findings indicate that dexamethasone can be potentially used in other autoimmune diseases to reprogram macrophages and other immune cells toward the anti-inflammatory phenotype.

Chronic low-grade inflammation involving macrophage activity promotes the development of another inflammatory disease, i.e., osteoarthritis (OA). In OA, like in other inflammatory diseases, macrophages play a dual and opposite role. On one hand, they are major players in the development of chronic inflammation and cartilage destruction, but they are also involved in cartilage remodeling and repair. This indicates that the ideal therapeutic strategy against macrophages should target pro-inflammatory and preserve anti-inflammatory macrophages. Mushenkova et al. [10] studied the role of the heterogeneity of macrophages in OA. They found that the inflammatory processes in OA depend on macrophage heterogeneity and plasticity in changing phenotypes, which differ between individual patients and the stage of the disease. At present, there is still no clear classification of functional macrophage subpopulations established in OA. Authors suggest that the scRNA sequencing methods can help in defining details of macrophage heterogeneity, but there is also a need for the identification and validation of biomarkers specific for distinct macrophage subgroups.

Macrophages are not only involved in inflammatory diseases but also in the homeostasis and aging processes. Aging is associated with low-grade inflammation harmful to physiological functions. Such systemic inflammation is handled by liver macrophages which produce cytokine and scavenge bacteria from the circulation preventing the infection of other organs. Thus, with progressing aging, the number of liver macrophages increases. Liver macrophages, among other functions, are responsible for iron homeostasis by phagocyting nonfunctional erythrocytes, recovering iron from them, and returning it into circulation. Iron was suggested to shift macrophage phenotype to the M2 subtype, but its effect on macrophage proliferation was unknown. Bloomer [11] studied how macrophage phenotype, number, and accumulation of iron in the liver change with age. He showed in the rat model that aging increases the number of M1 (CD68+) and M2 (CD163+) macrophages in the liver and that both macrophage subtypes stored iron. This study concluded that iron storage does not modify macrophage number or phenotype. Therefore, macrophages can store excess iron without proliferating or shifting their phenotype.

The heterogeneity of macrophage type and function increases to another level of complexity by the ability of macrophages to fuse with each other or with different cell types.

Kloc et al. [5] describe how the fusion of monocyte-macrophage lineage cells creates syncytial multinucleated giant cells (MGCs) (Figure 1). Examples of MGCs are the bone-resorbing osteoclasts (OCs), the pathogen-induced Langhans giant cells (LGCs), and foreign body-induced giant cells (FBGCs). These giant cells have increased phagocytic and other functional abilities in comparison to individual macrophages. MGCs are also present in various tumors where they phagocyte cancer cells and debris. For example, MGCs are present in 31% of esophageal cancers where they participate in the anti-cancer immune response. Studies showed that the presence of MGCs in the tumor decreases metastases and improves patient survival prognosis [12]. MGCs also form after the fusion of virally infected cells with non-infected ones. These giant cells serve as the reservoir of the virus and are the long-lasting source of virus dissemination (Figure 1).

One of the important aspects relevant to macrophage studies addressed by Haider et al. [13] is how or if different cell preservation techniques influence the quantitative and functional analyses of immune cells. The authors analyzed how the cryopreservation of mononuclear cells (MNCs), i.e., blood cells with a single, round nucleus, such as lymphocytes and monocytes, taken from 15 individuals, affected cell quantity and inflammatory responses. They tested four cryopreservation media and freeze-thawing protocols and found that they not only cause differences in cell viability and recovery but also differently affect the inflammatory response of MNCs. These results indicate that any study of cryopreserved samples needs careful analysis and controls to eliminate biases and artifacts.

## Figures and Tables

**Figure 1:**
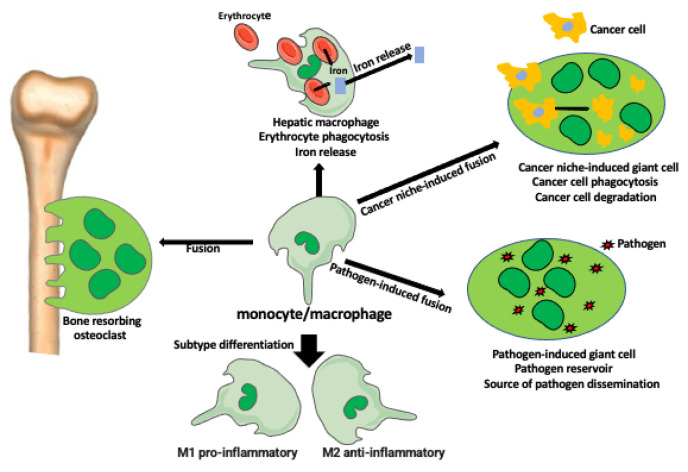
Diversity of macrophage functions.
